# Combined Antiseizure Efficacy of Cannabidiol and Clonazepam in a Conditional Mouse Model of Dravet Syndrome

**DOI:** 10.33696/neurol.2.040

**Published:** 2021

**Authors:** Shu-Hui Chuang, Ruth E. Westenbroek, Nephi Stella, William A. Catterall

**Affiliations:** 1 Department of Pharmacology, University of Washington, Seattle, Washington 98195, USA; 2 Department of Psychiatry & Behavioral Sciences, University of Washington, Seattle, Washington 98195, USA

**Keywords:** Dravet syndrome, Na_v_1.1, Scn1a, Febrile seizures, Cannabidiol, Benzodiazepines, Combination therapy

## Abstract

Dravet Syndrome (DS) is a severe childhood epilepsy caused by heterozygous loss-of-function mutations in the *SCN1A* gene encoding brain type-I voltage-gated sodium channel Na_v_1.1. DS is a devastating disease that typically begins at six to nine months of age. Symptoms include recurrent intractable seizures and premature death with severe neuropsychiatric comorbidities, including hyperactivity, sleep disorder, anxiety-like behaviors, impaired social interactions, and cognitive deficits. There is an urgent unmet need for therapeutic approaches that control and cure DS, as available therapeutic interventions have poor efficacy, intolerance, or other side effects. Here we investigated the therapeutic potential of combining the benzodiazepine clonazepam (CLZ) with the nonpsychotropic phytocannabinoid cannabidiol (CBD) against thermally induced febrile seizures in a conditional mouse model of DS. Our results show that a low dose of CLZ alone or combined with CBD elevated the threshold temperature for the thermal induction of seizures. Combination of CLZ with CBD significantly reduced seizure duration compared to the vehicle or CLZ alone, but did not affect seizure severity, indicating potential additive actions of CLZ and CBD on the duration of seizures. Our findings provide preclinical evidence supporting combination therapy of CLZ and CBD for treatment of febrile seizures in DS.

## Introduction

Dravet syndrome (DS) is an intractable childhood epilepsy disorder affecting one in 15,000 to 20,000 births [1]. It is caused by *de novo* heterozygous loss-of-function mutations in the *SCN1A* gene encoding the brain type-I voltage-gated sodium channel Na_v_1.1. The onset typically occurs at six to nine months of age with seizures triggered by elevated body temperature caused by fever, a warm bath, or a hot day. Febrile seizures are followed by recurrent spontaneous seizures that are resistant to current medications. Comorbidities including hyperactivity, sleep disorder, anxiety-like behaviors, deficits in social interaction and cognitive functions, and sudden death are seen in most DS patients [1]. There is an urgent unmet need to develop novel treatments for DS, as current therapies are limited by poor efficacy, intolerance, and side effects.

Current medications for treatment of seizures associated with DS include the first-line antiepileptic drugs clobazam and valproic acid plus second-line treatments such as stiripentol, topiramate, and ketogenic diet [2]. Cannabidiol (CBD) oral solution was approved to treat seizures related to DS and Lennox-Gastaut syndrome in patients two years of age or older, when given in addition to standard-of-care medications [3]. This nonpsychoactive phytocannabinoid holds promise to become a novel therapeutic modality to treat seizures and related conditions [4–8]. CBD attenuates seizures and improves social and cognitive deficits in DS mice through the modulation of GABAergic neurotransmission [4]. However, recent studies suggest that CBD treatment displays low efficacy and has risk of seizure aggravation when used as single agent, suggesting a need to explore combinations with standard-of-care medications [9].

Clonazepam (CLZ) is a positive allosteric modulator of GABA_A_ receptors that potentiates responses to GABA without opening the GABA_A_ receptor chloride channel itself. CLZ is a first-line benzodiazepine for treatment of acute seizures, but it is not suitable for long-term treatment to prevent seizures due to tolerance. Using a well-established *Scn1a*^+/−^ mouse model of DS [10], we found that low-dose CLZ improves impaired social interaction and contextual learning without sedative side effects [11]. Here we hypothesized that combination treatment of CLZ with CBD may exert additive therapeutic efficacy against thermally induced seizures. To test this hypothesis, we used a conditional DS mouse model in which one allele of the *Scn1a* gene was deleted in all epiblast-derived somatic cells using Cre recombinase expressed under the *Meox2* promoter [12,13].

## Material and Methods

### Animals

Animal experiments were performed according to guidelines established in the National Institutes of Health Guide for Care and Use of Laboratory Mice and in compliance with a protocol approved by the Institutional Animal Care and Use Committee of the University of Washington, Seattle. The conditional mouse model of DS was previously described [13]. Briefly, *Scn1a* mutant mice generated by targeted deletion of exon 25 [12] were maintained on a C57BL/6 background (Jackson Laboratories, Bar Harbor, ME). Conditional deletion of *Scn1a* in all epiblast-derived somatic cells was achieved by crossing a floxed *Scn1a* mouse with a Meox2-Cre mouse in the same genetic background (F/+:Meox2-Cre^+^ DS Mice). Both male and female offspring were used for experiments.

### Thermal induction of seizures

Febrile seizures were thermally induced on postnatal days P21-P28 using established methods [14]. Core body temperature was continuously monitored with a rectal temperature probe and controlled to ± 0.3°C with a feedback temperature controller (TCAT2DF; Physitemp) and heat lamp. Temperature was elevated 0.5°C in 2-min intervals until a generalized tonic-clonic seizure (GTC) occurred or 41°C was reached. The GTC threshold temperature of each animal was recorded during thermal induction and verified in videos. The duration and severity of each seizure were determined from video recordings. The severity of seizures was evaluated according to Racine’s scale [15].

### Drugs

CLZ (Sigma) was diluted in a saline solution containing 0.5% methylcellulose (Sigma). CBD (Cayman) was dissolved in a vehicle containing 1% ethanol:1% cremophore:18% saline. CLZ at 0.0625 mg/kg and CBD at 100 mg/kg were administered intraperitoneally 0.5 and 1 h before seizure induction, respectively.

### Statistical analysis

Data are presented as mean ± SEM. An unpaired Student t-test was used to compare groups. All analyses were performed using GraphPad Prism 8 (Graphpad Software Inc., La Jolla, California, USA). Statistical significance was set at p<0.05; n=10 – 12 per group.

## Results

We used a newly developed conditional mouse model of DS [13] to investigate the combined therapeutic efficacy of CLZ and CBD against thermally induced seizures. We treated mice with 0.0625 mg/kg of CLZ, which rescued the impaired social interaction and fear learning in DS mice without sedative effects [11]. We combined CLZ with CBD at 100 mg/kg, which elicits robust antiseizure activity [4]. To model febrile seizures, we used a thermal induction protocol mimicking fever in which core body temperature was elevated 0.5°C every 2 min until a GTC seizure was induced or 41°C was reached.

### CLZ alone or combined with CBD elevates the threshold for thermal induction of seizures

**Figure 1:**
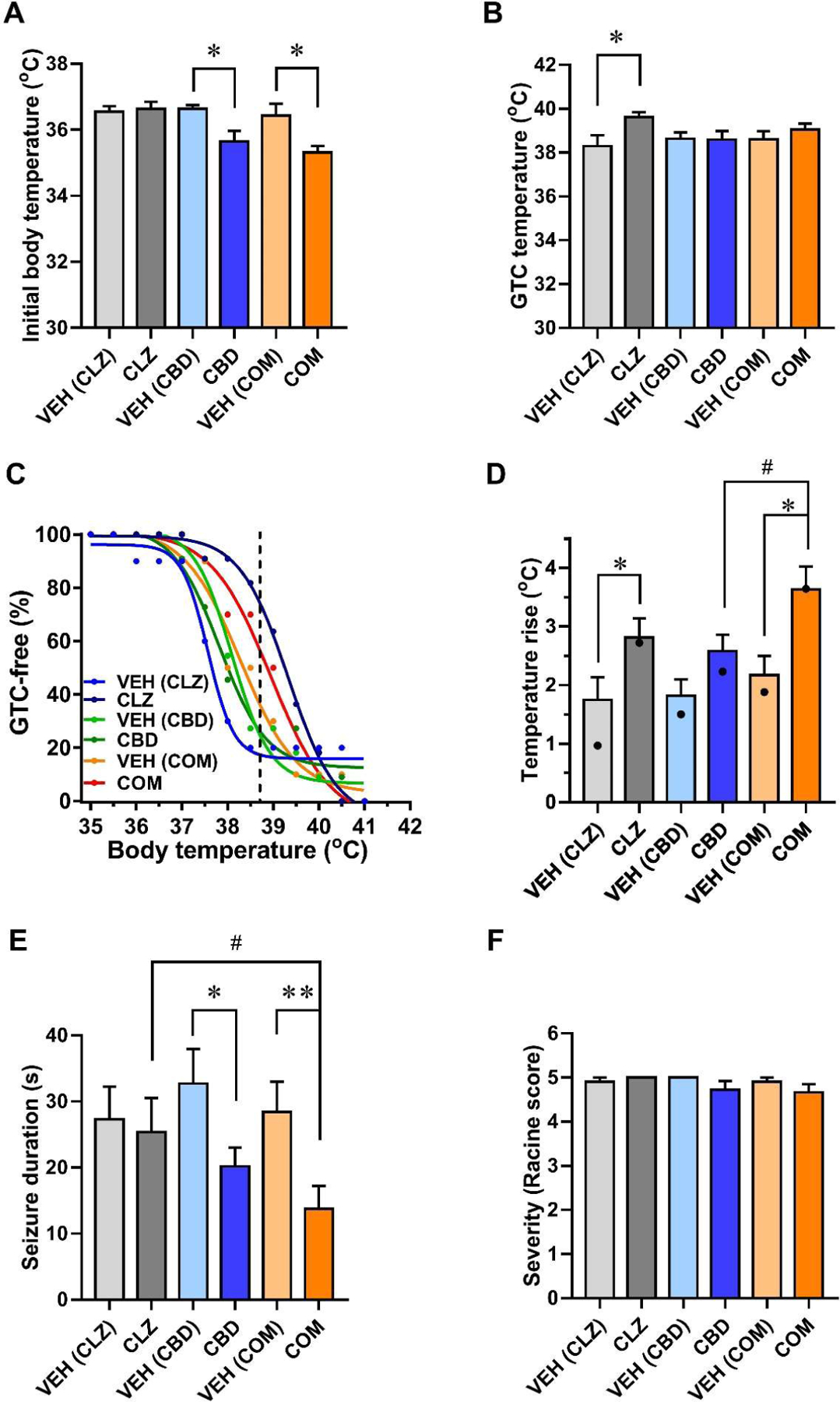
Combination treatment with CBD and CLZ elevates the threshold temperature for thermal induction of seizures and additively decreases the duration of febrile seizures in DS mice. **(A)** The baseline body temperature of CLZ-, CBD-, or combination (COM) treated DS mice and their respective controls. Body temperature (°C): VEH (CLZ), 36.6 ± 0.17; CLZ, 36.6 ± 0.18; VEH (CBD), 36.6 ± 0.11; CBD, 35.7 ± 0.33; VEH (COM), 36.4 ± 0.36; COM, 35.3 ± 0.20 (*, p<0.05 for CBD and COM vs. respective VEH; n = 10 – 12). **(B)** The threshold temperature for thermal induction of generalized tonic-clonic (GTC) seizures in DS mice. GTC temperature (°C): VEH (CLZ), 38.3 ± 0.50; CLZ, 39.4 ± 0.27; VEH (CBD), 38.6 ± 0.29; CBD, 38.6 ± 0.40; VEH (COM), 38.6 ± 0.37; COM, 39.0 ± 0.28 (*, p < 0.05 CLZ vs. VEH; n = 10 – 12). **(C)** Percentage of GTC-free mice during elevation of body temperature. Curves represent sigmoidal fits from GraphPad. The dashed line indicates the average threshold temperature for the thermal induction of seizures in controls (*, p<0.05 vs. the respective vehicle control; n = 10 – 12). (**D**) Increase in the core body temperature that causes febrile seizures in mice, determined by comparing the values in panels A and B. Black dots indicate the increase in core body temperature at which 50% of mice had seizures as estimated from the fit curves in panel C (*, p<0.05 vs. VEH; ^#^p<0.05 vs. CBD alone). CLZ vs. VEH (CLZ), p = 0.046; CBD vs. VEH (CBD), p = 0.073; COM vs. VEH (COM), p = 0.011; COM vs. CLZ, p = 0.125; COM vs. CBD, p = 0.048. **(E)** Seizure duration of CLZ-, CBD-, or combination- treated DS mice and their respective controls. Seizure duration (s): VEH (CLZ), 27.3 ± 5.0; CLZ, 25.4 ± 5.2; VEH (CBD), 32.7 ± 5.2; CBD, 20.2 ± 2.8; VEH (COM), 28.4 ± 4.6; COM, 13.8 ± 3.5 (*, p<0.05 vs. VEH; ^#^p<0.05 vs. CLZ alone; n = 10 – 12). **(F)** Severity (Racine Score). VEH: vehicles for each treatment as indicated; CLZ: clonazepam; CBD: cannabidiol; COM: combination. CBD at 100 mg/kg and CLZ at 0.0625 mg/kg were administered 1 and 0.5 h before thermal induction of seizures, respectively.

CBD and combination (COM)-treated mice exhibited body temperatures ~1° lower than untreated controls, whereas low-dose CLZ did not alter body temperature (Figure 1A). When core body temperature was controlled and increased slowly and progressively, a low dose of CLZ significantly elevated the threshold temperature for thermal induction of seizures (Figure 1B). Combination of CLZ and CBD also showed a trend toward increasing the threshold temperature for seizure induction, but the increase was smaller than treatment with CLZ alone (Figure 1B). With progressive elevation of body temperature, all mice tested gradually experienced seizures, and none remained seizure-free at 41°C (Figure 1C). CLZ alone or combined with CBD shifted the thermal induction curves to higher temperatures, indicating decreased seizure susceptibility (Figure 1C). Analyzing the effect of combined treatment on the increase in the core body temperature required to induce 50% of mice to have seizures using these temperature response curves showed a greater effect than with either drug alone (Figure 1D, COM, p<0.02; Figure 1D legend). Thus, CBD enhanced the effects of low-dose CLZ on thermal induction of seizures in these thermal induction experiments.

### Combined CLZ and CBD additively decreased duration of thermally induced seizures

We analyzed video recordings of seizure behavior to determine the duration and severity of seizures (Figures 1E and 1F). Although CLZ alone had no effect on either parameter, CBD alone significantly reduced seizure duration without affecting seizure severity (Figures 1E and 1F). Moreover, combination of CLZ and CBD resulted in a greater reduction in the duration of thermally evoked seizures compared to vehicle- or CLZ-treated mice, revealing additive beneficial effects of these compounds (Figure 1E). In contrast, these treatments did not affect seizure severity in DS mice (Figure 1F). Overall, our results demonstrate additive beneficial effects of combination treatment regimens using CLZ and CBD to better control thermally induced seizures in a conditional mouse model of DS.

## Discussion

The pathogenic mechanisms underlying DS include severely reduced sodium currents and action potential firing in GABAergic interneurons, causing hyperexcitability in neural circuits [10,12,16]. Pharmacological enhancement of GABAergic neurotransmission is a common approach for the control of seizures in DS patients [2]. Previously we found that combination of a GABA reuptake inhibitor, tiagabine, plus CLZ exert synergistic effects against thermally induced myoclonic and GTC seizures [17]. The synergistic response eliminates unwanted sedative effects, as lower doses were used to achieve effective protection against seizures. Evidently, combination drug therapies acting on both presynaptic (tiagabine) and postsynaptic (CLZ) components of GABAergic synapses may be beneficial for control of seizures in DS.

CBD reduces seizure duration in DS mice through a mechanism that is independent of the classical cannabinoid receptors CB1 and CB2, but may involve presynaptic GPR55 receptors, which are activated by lipid mediators [4]. In hippocampus, GPR55 is highly expressed in interneurons and excitatory neurons, where it controls presynaptic neurotransmitter release [18]. Inhibition of GPR55 prevents the beneficial effects of CBD on seizure duration in DS mice [4], suggesting that the effect of CBD may result from presynaptic antagonism of GPR55.

Although low-dose CLZ alone is not sufficient to exert strong protective effects against seizures, it improves social and learning deficits in DS mice [11]. Here we found that low-dose CLZ acts in an additive manner with CBD to reduce the thermal sensitivity for seizure induction and to reduce seizure duration through postsynaptic enhancement of GABAergic neurotransmission. This additive action may result from combining GABA-enhancing effects in both presynaptic and postsynaptic compartments of GABAergic synapses.

## Conclusions

Our results reveal additive benefits of combined treatment with CLZ and CBD in a well-validated conditional mouse model of DS. This combination therapy is also likely to improve other comorbidities such as deficits in social interaction and contextual learning without unwanted side effects. These findings provide a practical approach for the control and treatment of DS through combination therapy with agents acting presynaptically (CBD) and postsynaptically (CLZ) in a *Scn1a*^+/−^ mouse model. In the context of treatment of DS patients, our results support the possibility that seizure control could be achieved by adding CBD to standard treatment regimens and reducing the level of CLZ or clobazam, which may provide adequate seizure control and reduce the unwanted side effects of high-dose CLZ or clobazam on sedation, cognition, and social behavior.

## Highlights

- A low dose of clonazepam significantly increased the threshold temperature for thermal induction of seizures in Dravet syndrome mice
- Combined treatment with cannabidiol and clonazepam reduced thermal sensitivity and seizure duration in an additive manner
- Combined antiseizure therapy with cannabidiol and clonazepam is disclosed for the first time in a conditional mouse model of Dravet Syndrome

## Abbreviations:

CBD: Cannabidiol
CLZ: Clonazepam
COM: Combination
DS: Dravet Syndrome
GTC: Generalized Tonic-clonic Seizure
